# Preparation and Bioevaluation of Novel ^99m^Tc-Labeled Complexes with a 2-Nitroimidazole HYNIC Derivative for Imaging Tumor Hypoxia

**DOI:** 10.3390/ph14020158

**Published:** 2021-02-15

**Authors:** Qing Ruan, Qianqian Gan, Xuran Zhang, Si’an Fang, Junbo Zhang

**Affiliations:** Key Laboratory of Radiopharmaceuticals of Ministry of Education, College of Chemistry, Beijing Normal University, Beijing 100875, China; 201931150055@mail.bnu.edu.cn (Q.R.); 201731150054@mail.bnu.edu.cn (Q.G.); zhangxr@bnu.edu.cn (X.Z.); 201531150045@mail.bnu.edu.cn (S.F.)

**Keywords:** 2-nitroimidazole, technetium-99m, HYNIC, tumor, hypoxia

## Abstract

To develop novel ^99m^Tc-labeled single-photon emission computed tomography (SPECT) radiotracers for imaging hypoxia, a novel HYNICNM ligand (6-hydrazinonicotinamide (HYNIC) 2-nitroimidazole derivative) was designed and synthesized. It was radiolabeled with technetium-99m using tricine/trisodium triphenylphosphine-3,3′,3′′-trisulfonate (TPPTS), tricine/sodium triphenylphosphine-3-monosulfonate (TPPMS) and tricine as co-ligands to obtain [^99m^Tc]Tc-tricine-TPPTS-HYNICNM, [^99m^Tc]Tc-tricine-TPPMS-HYNICNM, and [^99m^Tc]Tc-(tricine)_2_-HYNICNM, respectively. The three technetium-99m complexes were radiolabeled in one step with a high yield (95%) and had good stability in saline and mouse serum. In vitro cellular uptake results showed that these complexes exhibited good hypoxic selectivity. The partition coefficient indicated that they were good hydrophilic complexes, and [^99m^Tc]Tc-tricine-TPPTS-HYNICNM displayed the highest hydrophilicity (−3.02 ± 0.08). The biodistribution in mice bearing S180 tumors showed that [^99m^Tc]Tc-tricine-TPPTS-HYNICNM exhibited higher tumor uptake (1.05 ± 0.27% IA/g); more rapid clearance from the liver, blood, muscle, and other non-target organs; and a higher tumor/non-target ratio, especially for the tumor/liver ratio (1.95), than [^99m^Tc]Tc-tricine-TPPMS-HYNICNM and [^99m^Tc]Tc-(tricine)_2_-HYNICNM. The results of single-photon emission computed tomography (SPECT) imaging studies of [^99m^Tc]Tc-tricine-TPPTS-HYNICNM were in accordance with the biodistribution results, which suggested that [^99m^Tc]Tc-tricine-TPPTS-HYNICNM is a promising agent for imaging tumor hypoxia.

## 1. Introduction

Hypoxia occurs in various solid tumors when the consumption of oxygen exceeds the supply of the bloodstream with uncontrolled tumor growth. It is regarded as a factor determining tumor aggressiveness, invasiveness, and therapy resistance, especially for radiotherapy and chemotherapy [1,2]. In addition to its direct impact on tumor therapy, hypoxia is associated with a number of molecular signaling pathways that influence tumor behavior. With the discovery progress of the hypoxia-inducible factor (HIF), the transcriptional regulation of hypoxia-induced genes has been widely acknowledged [3,4]. Therefore, it is important for tumor treatment to identify the oxygenation status of solid tumors [5].

Clinically, the gold standard for detecting tumor hypoxia is the oxygen needle electrode method, which can obtain relatively accurate measurement results of local oxygen concentrations and involves the insertion of a fine needle electrode into the accessible region in the tumor, which is invasive [6,7]. In comparison, nuclear imaging techniques, including single-photon emission computed tomography (SPECT) and positron emission tomography (PET) with hypoxia imaging agents, are more favorable for the clinical detection of tumor hypoxia due to their non invasion and visualization of biochemical activity in subjects.

Nitroimidazoles, including 2-,4-,5-nitroimidazole, are considered effective pharmacophores for detecting tumor hypoxia because under hypoxic conditions, nitroimidazoles are reduced by cellular oxidoreductases, and the intermediates irreversibly bind to intracellular components in hypoxic regions [8]. In particular, 2-nitroimidazole, with a more positive single-electron reduction potential (SERP), has more potential for reduction in cells and wider use for detecting hypoxia [9].

Several SPECT and PET radiotracers with nitroimidazole have been reported to date and have attracted attention [10,11,12,13,14,15,16,17,18,19,20,21]. [^18^F]FMISO is one of the most widely used PET radiotracers for imaging hypoxia [22]. However, because of its relatively low uptake in hypoxic tumor sites and low target-to-background ratio in PET imaging, it is insufficient to be considered an ideal hypoxia imaging agent [5,23,24]. Therefore, developing a novel hypoxia tracer with excellent performance is necessary and urgently needed for clinical treatment.

Technetium-99m has been the most widely used SPECT isotope because it possesses ideal nuclide properties (*t*_1/2_ = 6 h, Eg = 140 keV) and rich coordination chemistry with various oxidation states and donor atom sets [25]. In addition, its in-house availability and reasonable cost have resulted in wide clinical use, especially in many developing countries and less-developed regions [13,14,15,16].

Hydrazinonicotinamide (HYNIC), which was first reported by Abrams et al. [26] in the early 1990s, is an attractive bifunctional chelator that is widely used to couple with technetium-99m and form stable radiolabeled complexes [27,28,29,30,31,32]. In addition, with different co-ligands, such as tricine, ethylenediamine-*N,N’*-diacetic acid (EDDA) and triphenylphosphine sulfonates (TPPTS, trisodium triphenylphosphine-3,3′,3′′-trisulfonate; TPPDS, disodium triphenylphosphine-3,3′-disulfonate; and TPPMS, sodium triphenylphosphine-3-monosulfonate), these radiotracers exhibited obvious diverse results in terms of biodistribution and lipophilicity, and many studies have been reported to date [33,34,35].

We previously reported a series of ^99m^Tc-labeled complexes with nitroimidazole isocyanide or xanthate [13,36,37]. They all exhibited good hypoxic selectivity and favorable accumulation in tumors. However, quite a high uptake in the abdomen, especially in the liver, affected the quality of SPECT imaging and limited their clinical application to a certain extent. In this work, we synthesized a 2-nitroimidazole HYNIC derivative and radiolabeled it with technetium-99m using tricine, tricine/TPPTS, and tricine/TPPMS as co-ligands to evaluate their potential as SPECT imaging probes for tumor hypoxia.

## 2. Results

### 2.1. Synthesis of HYNICNM

**Scheme 1:**
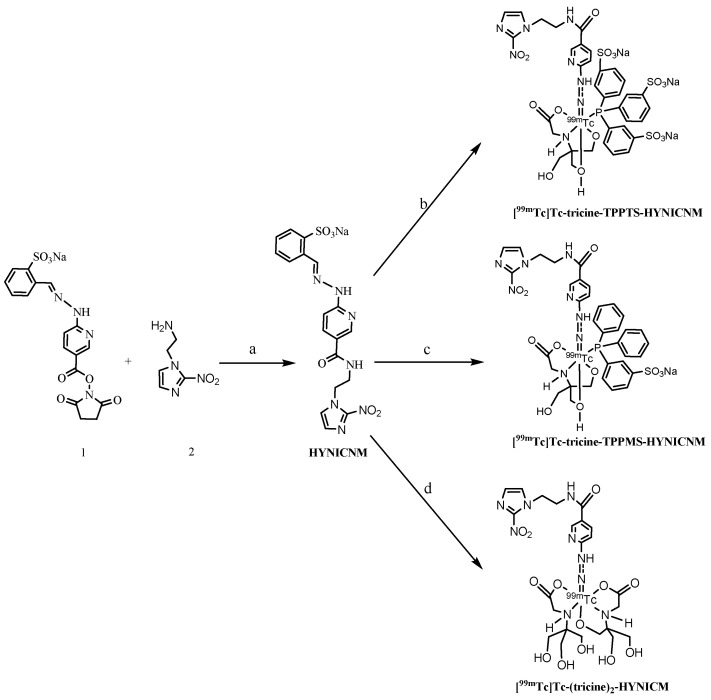
The synthesis and speculative structures of [^99m^Tc]Tc-tricine-TPPTS-HYNICNM, [^99m^Tc]Tc-tricine-TPPMS-HYNICNM, and [^99m^Tc]Tc-(tricine)_2_-HYNICNM. Reagents and conditions: (**a**) DMF, TEA, reflux, 4 h; (**b**) Trisodium triphenylphosphine-3,3′,3′′-trisulfonate (TPPTS), tricine, SnCl_2_·2H_2_O, acetate buffer solution (pH 5, 0.5 M), [^99m^Tc]NaTcO_4_, 100 °C, 0.5 h; (**c**) Sodium triphenylphosphine-3-monosulfonate (TPPM, tricine, SnCl_2_·2H_2_O, acetate buffer solution (pH 5, 0.5 M), [^99m^Tc]NaTcO_4_, 100 °C, 0.5 h; (**d**) tricine, SnCl_2_·2H_2_O, acetate buffer solution (pH 5, 0.5 M), [^99m^Tc]NaTcO_4_, 100 °C, 0.5 h.

The HYNIC-containing active ester (Compound **1**) was synthesized in three steps [35], and the amino derivative of 2-nitroimidazole (Compound **2**) was prepared via two steps from 2-nitroimidazole [13], which are shown in the Supplementary Material in detail. The ligand, HYNICNM, was synthesized by Compound **1** and Compound **2** in one step (yield, 67%), as shown in Scheme 1, and the final product was identified by ^1^H NMR, ^13^C NMR, IR, and HR-MS (Figures S3–S6), indicating that the target ligand was successfully synthesized as our proposed structure.

### 2.2. Radiolabeling and Quality Control

The ligand HYNICNM can be easily labeled in one pot by the reaction of pertechnetate with stannous chloride as a reducing agent in the presence of excess tricine to obtain [^99m^Tc]Tc-(tricine)_2_-HYNICNM. By adding TPPTS or TPPMS to the reaction system together with tricine, a ternary ligand system complex, [^99m^Tc]Tc-tricine-TPPTS-HYNICNM or [^99m^Tc]Tc-tricine-TPPMS-HYNICNM, was also produced.

**Figure 1:**
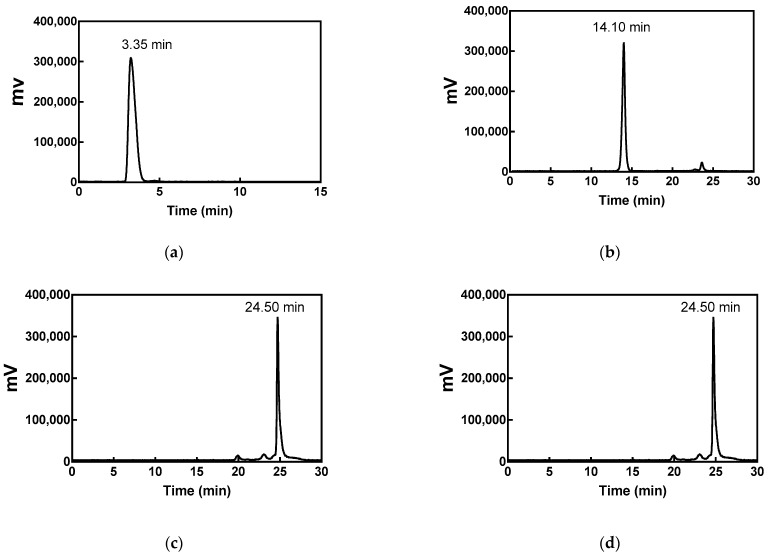
HPLC patterns of ^99m^Tc-complexes: (**a**) [^99m^Tc]NaTcO_4_; (**b**) [^99m^Tc]Tc-tricine-TPPTS-HYNICNM; (**c**) [^99m^Tc]Tc-tricine-TPPMS-HYNICNM; (**d**) [^99m^Tc]Tc-(tricine)_2_-HYNICNM.

As assessed by HPLC, the radiochemical purities of [^99m^Tc]Tc-tricine-TPPTS-HYNICNM, [^99m^Tc]Tc-tricine-TPPMS-HYNICNM, and [^99m^Tc]Tc-(tricine)_2_-HYNICNM were all over 95%, suggesting that these complexes can be used for in vitro and in vivo studies without further purification. The retention times were 14.10 min, 24.50 min, and 22.85 min, respectively (Figure 1), which were separated well from those of [^99m^Tc]NaTcO_4_ (3.35 min), [^99m^Tc]Tc-Tricine (3.15 min), and [^99m^Tc]Tc-tricine-TPPTS (4.07 min in Figure S7). To determine the radiochemical purity quickly, thin layer chromatography (TLC) can be routinely used. In the developing solvent of saline, [^99m^Tc]Tc-tricine-TPPTS-HYNICNM moved to the solvent front (R_f_ = 0.8–1.0), [^99m^Tc]TcO_2_·nH_2_O remained at the origin (R_f_ = 0–0.1), while [^99m^Tc]NaTcO_4_ moved to the middle position (R_f_ = 0.5–0.7). TLC results showed that the radiochemical purity of [^99m^Tc]Tc-tricine-TPPTS-HYNICNM was over 95%.

### 2.3. In Vitro Stability Study and Partition Coefficient

The radiochemical purities of [^99m^Tc]Tc-tricine-TPPTS-HYNICNM, [^99m^Tc]Tc-tricine-TPPMS-HYNICNM, and [^99m^Tc]Tc-(tricine)_2_-HYNICNM were still greater than 90% in saline at room temperature and in mouse serum at 37 °C after 6 h (Figures S8–S10), which indicated their good in vitro stability. The partition coefficient (log P) values of [^99m^Tc]Tc-tricine-TPPTS-HYNICNM, [^99m^Tc]Tc-tricine-TPPMS-HYNICNM, and [^99m^Tc]Tc-(tricine)_2_-HYNICNM were −3.02 ± 0.08, −0.76 ± 0.03, and −1.73 ± 0.02, respectively, suggesting that the complexes were hydrophilic. Among them, [^99m^Tc]Tc-tricine-TPPTS-HYNICNM exhibited stronger hydrophilicity than the others, which indicated that the co-ligand plays an important role in the hydrophilicity of the technetium-99m complexes.

### 2.4. In Vitro Cellular Uptake

**Figure 2:**
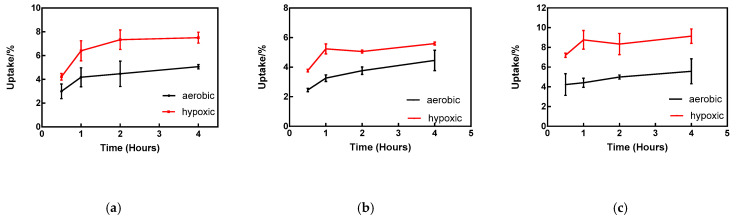
Cellular uptake of ^99m^Tc-complexes: (**a**) [^99m^Tc]Tc-tricine-TPPTS-HYNICNM; (**b**) [^99m^Tc]Tc-tricine-TPPMS-HYNICNM; (**c**) [^99m^Tc]Tc-(tricine)_2_-HYNICNM.

The cellular uptake of [^99m^Tc]Tc-tricine-TPPTS-HYNICNM, [^99m^Tc]Tc-tricine-TPPMS-HYNICNM, and [^99m^Tc]Tc-(tricine)_2_-HYNICNM was carried out under hypoxic and aerobic conditions in the S180 cell line, which is a representative cell for hypoxic selectivity and hypoxia model of solid tumors [37,38,39] (Figure 2). As determined by Student’s *t*-test, the uptake of the complexes under hypoxic conditions and aerobic conditions had significant differences (independent, two-tailed, *p* < 0.05) at each time point, suggesting that they had good hypoxia selectivity.

### 2.5. Biodistribution Studies

**Figure 3:**
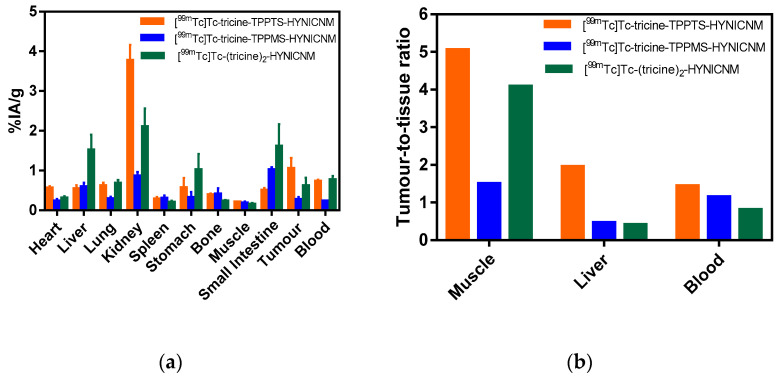
(**a**) Comparisons of biodistribution of [^99m^Tc]Tc-tricine-TPPTS-HYNICNM, [^99m^Tc]Tc-tricine-TPPMS-HYNICNM, and [^99m^Tc]Tc-(tricine)_2_-HYNICNM at 2 h post-injection (*n* = 5). (**b**) Comparisons of the tumor-to-blood, tumor-to-muscle, and tumor-to-liver ratios of [^99m^Tc]Tc-tricine-TPPTS-HYNICNM, [^99m^Tc]Tc-tricine-TPPMS-HYNICNM, and [^99m^Tc]Tc-(tricine)_2_-HYNICNM at 2 h post-injection (*n* = 5).

[^99m^Tc]Tc-tricine-TPPTS-HYNICNM, [^99m^Tc]Tc-tricine-TPPMS-HYNICNM, and [^99m^Tc]Tc-(tricine)_2_-HYNICNM were evaluated in Kunming female mice to determine their ability to target hypoxic tumors. The biodistribution results of [^99m^Tc]Tc-tricine-TPPTS-HYNICNM are presented in Table 1, and comparisons of the three technetium-99m complexes are shown in Figure 3 and Table S1. As demonstrated in Figure 3a, the uptake of the kidney was relatively high for the three complexes at 2 h post-injection, indicating that their main excretion route is urinary. Compared with [^99m^Tc]Tc-tricine-TPPTS-HYNICNM, the uptake value in the small intestine of [^99m^Tc]Tc-tricine-TPPMS-HYNICNM (1.02 ± 0.06% IA/g) and [^99m^Tc]Tc-(tricine)_2_-HYNICNM (1.61 ± 0.56% IA/g) was much higher, and the liver uptake of [^99m^Tc]Tc-(tricine)_2_-HYNICNM (1.52 ± 0.38% IA/g) was also much higher than the others, which suggests that the two radiotracers are partly excreted via the hepatic and gastrointestinal tract.

At 2 h post-injection, [^99m^Tc]Tc-tricine-TPPTS-HYNICNM exhibited a higher tumor uptake (1.05 ± 0.27% IA/g) than [^99m^Tc]Tc-tricine-TPPMS-HYNICNM (0.27 ± 0.06% IA/g) and [^99m^Tc]Tc-(tricine)_2_-HYNICNM (0.62 ± 0.20% IA/g). With the rapid clearance from liver, lungs, blood, muscle, and other non-target tissues, [^99m^Tc]Tc-tricine-TPPTS-HYNICNM also exhibited a significantly higher tumor/non-target ratio than that of [^99m^Tc]Tc-tricine-TPPMS-HYNICNM and [^99m^Tc]Tc-(tricine)_2_-HYNICNM, especially the tumor/muscle ratio (5.05), tumor/liver ratio (1.95), and tumor/blood ratio (1.44).

### 2.6. SPECT/CT Imaging Studies

**Figure 4:**
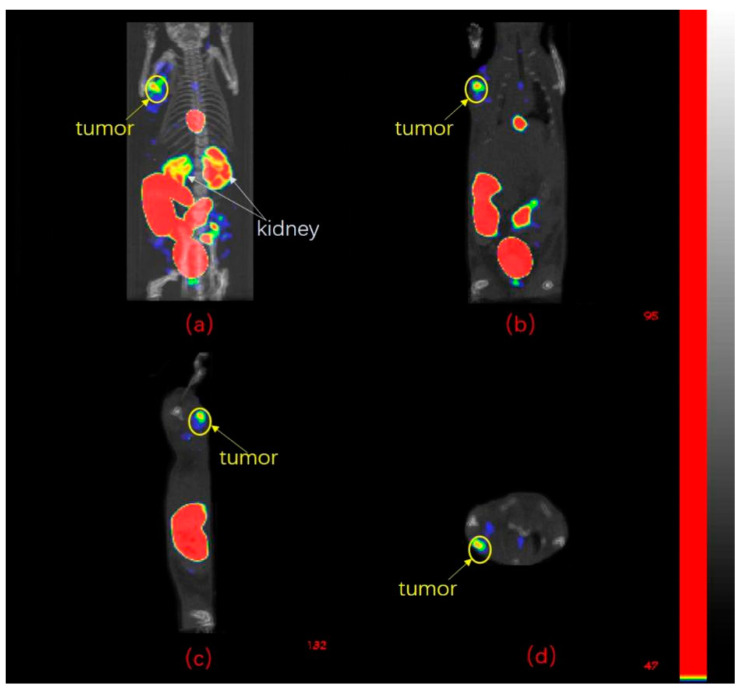
Single-photon emission computed tomography (SPECT)/CT images of [^99m^Tc]Tc-tricine-TPPTS-HYNICNM in Kunming female mice bearing S180 tumors at 2 h post-injection: (**a**) visual imaging; (**b**) coronal section; (**c**) sagittal section; (**d**) transverse section.

According to the biodistribution results, [^99m^Tc]Tc-tricine-TPPTS-HYNICNM was selected for further SPECT/CT imaging study, and the imaging is shown in Figure 4. The S180 tumor at the left front armpit of mice was clearly visible at 2 h post-injection. However, a high accumulation of radioactivity was also observed in the gallbladder, kidneys, urinary bladder, and large intestine.

## 3. Discussion

Nitroimidazole is the most widely used pharmacophore for hypoxia imaging because it can differentiate hypoxic and normoxic tissue. The potential for targeting hypoxia mostly depends on the single-electron reduction potential (SERP); the more positive the SERP value of a nitroimidazole is, the better its reduction potential of enzymes in aerobic cells [25]. Therefore, 2-nitroimidazole with the highest SERP value has become the most popular pharmacophore for the development of hypoxia probes compared with 4-nitroimidazole and 5-nitroimidazole.

For SPECT imaging, technetium-99m is still a very good choice due to its ideal nuclide properties, availability, and affordability. To covalently connect technetium-99m and 2-nitroimidazole to form a stable labeled compound for tumor hypoxia, it is important to select a suitable bifunctional chelator. With various bifunctional chelators, the biochemical properties of the radiotracers were obviously different. In our previous study, xanthate, a bidentate σ donor used for the preparation of [^99m^Tc][TcN]^2+^ and [^99m^Tc][TcO]^3+^ complexes, and isocyanide, a strongly monodentate coordination ligand that can form six-coordinated complexes with [^99m^Tc][Tc(I)]^+^ and [^99m^Tc][Tc(CO)_3_(OH_2_)_3_] cores, were used as bifunctional chelators for a series of ^99m^Tc-labeled complexes. Most of the complexes, such as [^99m^Tc]Tc-2c (a ^99m^Tc-labeled complex with 2-nitroimidazole isocyanide) and [^99m^Tc]TcO-MNXT (a [^99m^Tc][TcO]^3+^ labeled 5-nitroimidazole xanthate derivative), exhibited favorable accumulation in tumors and relatively high tumor/muscle ratios ([^99m^Tc]Tc-2c: 5.05; [^99m^Tc]TcO-MNXT: 6.13 at 2 h post-injection)[13,37]. However, the appreciable uptake in the abdomen, especially in the liver ([^99m^Tc]Tc-2c: 4.10 ± 0.55% IA/g; [^99m^Tc]TcO-MNXT: 8.61 ± 2.11% IA/g at 2 h post-injection), is a disadvantage of the complexes; thus, choosing the HYNIC group, a bifunctional linker with better hydrophilicity, seems to be a suitable solution to reduce non-target uptake.

However, the free hydrazine group of HYNIC is not stable in an aqueous solution and can react with other groups. Joyard et al. [40] prepared two 2-nitroimidazole HYNIC derivatives radiolabeled with technetium-99m, but decomposition of the ligands occurred, and they proposed a self-redox process between 2-nitroimidazole and HYNIC ligands. In addition, the hydrazine group can react with aldehydes and ketones, which may leach out from various rubber and plastic materials, to form various hydrazone impurities [33]. Therefore, a 2-sulfonatobenzaldehyde group was selected as the protecting group to block the reaction of the hydrazine moiety. Moreover, it is sufficiently labile to undergo hydrolysis under labeling conditions (pH = 5) to form free hydrazine. Since the HYNIC group can occupy only one site in the technetium coordination sphere, a co-ligand, such as tricine, is needed. To our delight, we demonstrated that [^99m^Tc]Tc-(tricine)_2_-HYNICNM, with the presence of the many isomeric forms [28], exists as nearly a single species in the radio-HPLC pattern, and it also exhibited stability in saline and mouse serum for 6 h. As technetium-99m has a distorted octahedral coordination geometry and HYNICNM occupies only one binding site, it requires at least two tricine ligands to complete the coordination sphere, and the proposed structure of [^99m^Tc]Tc-(tricine)_2_-HYNICNM is also supported by the work of Abrams et al. and Liu et al. [41,42]. As tricine is a weakly chelating co-ligand, one of the ligands can be exchanged by a strong ligand to bind technetium-99m. TPPTS and TPPMS are monodentate phosphines that produce a unique and versatile ternary ligand system with tricine and HYNICNM ligands to obtain [^99m^Tc]Tc-tricine-TPPTS-HYNICNM and [^99m^Tc]Tc-tricine-TPPMS-HYNICNM, respectively. The radiochemical purities of [^99m^Tc]Tc-tricine-TPPTS-HYNICNM and [^99m^Tc]Tc-tricine-TPPMS-HYNICNM were over 95%, and no obvious decomposition or dissociation was detected in saline at room temperature or in mouse serum at 37 °C for 6 h. In fact, we performed labeling using SnCl_2_ and a mixture of co-ligands ethylenediamine-*N,N’*-diacetic acid (EDDA) /tricine according to the literature [34,41]. However, a large number of radiolabeling methods have failed to provide a clean radio-HPLC chromatogram.

The hypoxic selectivity of [^99m^Tc]Tc-tricine-TPPTS-HYNICNM, [^99m^Tc]Tc-tricine-TPPMS-HYNICNM, and [^99m^Tc]Tc-(tricine)_2_-HYNICNM was measured by cellular uptake in the S180 cell line, which is a representative cell for hypoxic selectivity and hypoxia model of solid tumors [37,38,39]. As shown in Figure 4, the significantly higher uptake values under hypoxic conditions than under aerobic conditions provided evidence for the obvious hypoxic selectivity.

As we anticipated, [^99m^Tc]Tc-tricine-TPPTS-HYNICNM (log P = −3.02 ± 0.08), [^99m^Tc]Tc-tricine-TPPMS-HYNICNM (log P = −0.76 ± 0.03), and [^99m^Tc]Tc-(tricine)_2_-HYNICNM (log P = −1.73 ± 0.02) were all hydrophilic complexes. [^99m^Tc]Tc-tricine-TPPTS-HYNICNM showed significantly more hydrophilicity than [^99m^Tc]Tc-tricine-TPPMS-HYNICNM because TPPTS with two more sulfonic groups was more water-soluble than TPPMS, and the hydrophilicity of [^99m^Tc]Tc-(tricine)_2_-HYNICNM was in between those of the other two complexes.

In the biodistribution studies, the maximum uptake values of the radiotracer were observed at 30 min after injection, and the highest tumor-to-organ ratios and significant excretion of the technetium-99m complexes through the kidneys were observed at 2 h after injection. Among the complexes, [^99m^Tc]Tc-tricine-TPPTS-HYNICNM exhibited the highest kidney uptake, and this finding is considered to be associated with their hydrophilicity. For [^99m^Tc]Tc-tricine-TPPMS-HYNICNM, comparatively low tumor accumulation (0.27 ± 0.06% IA/g at 2 h post-injection) and high small intestine (1.02 ± 0.06% IA/g) and kidney (0.86 ± 0.10% IA/g) uptake were the main drawbacks for tumor imaging. [^99m^Tc]Tc-(tricine)_2_-HYNICNM exhibited obvious tumor uptake (0.62 ± 0.20% IA/g) at 2 h post-injection, and with rapid clearance from muscle, it showed a relatively high tumor/muscle ratio (4.08). However, the high uptake of non-target organs, such as the liver (1.52 ± 0.38% IA/g), kidney (2.10 ± 0.46% IA/g), stomach (1.02 ± 0.40% IA/g), and small intestine (1.61 ± 0.56% IA/g), will interfere with the imaging of abdominal tumors. Compared with [^99m^Tc]Tc-tricine-TPPMS-HYNICNM and [^99m^Tc]Tc-(tricine)_2_-HYNICNM, [^99m^Tc]Tc-tricine-TPPTS-HYNICNM displayed the highest tumor uptake (2.04 ± 0.22% IA/g at 0.5 h post-injection and 1.05 ± 0.27% IA/g at 2 h post-injection) and the most admirable tumor/liver (1.95), tumor/muscle (5.05), and tumor/blood (1.44) ratios at 2 h post-injection. TPPTS, the water-soluble co-ligand, increased the excretion from muscle, blood, liver, and other non-target tissues and reduced background interference for imaging. High liver uptake and a relatively low tumor/liver ratio are common disadvantages for most nitroimidazole radiotracers for imaging tumor hypoxia. For example, the tumor-to-liver ratio of [^18^F]FMISO is merely 0.82 in EMT-6 tumor-bearing mice at 120 min post-injection [11], which is less than half the ratio of [^99m^Tc]Tc-tricine-TPPTS-HYNICNM (1.95). Compared to [^18^F]FMISO, as [^99m^Tc]Tc-tricine-TPPTS-HYNICNM showed extremely low uptake in other major organs, it also displayed a higher tumor to non-targeted ratios (tumor/muscle: 5.05 vs. 1.45 at 2 h post-injection). Although with different varieties of mice, the ratios can show the superiority of [^99m^Tc]Tc-tricine-TPPTS-HYNICNM to a certain extent.

Because of the admirable biodistribution performance, [^99m^Tc]Tc-tricine-TPPTS-HYNICNM was selected for further SPECT/CT imaging study. As predicted, [^99m^Tc]Tc-tricine-TPPTS-HYNICNM imaging showed obvious tumor uptake. In particular, quite low uptake in the liver is one of its greatest advantages; thus, the purpose of the experiment was achieved. However, the uptakes of kidneys, urinary bladder, gallbladder, large intestine are evident, which indicates that the urinary and intestinal tract are the major routes of excretion. Similar imaging results can also be found in the other groups’ studies [43,44]. Considering the issue of toxicity, we performed a further abnormal toxicity study of [^99m^Tc]Tc-tricine-TPPTS-HYNICNM. None of five mice showed abnormality or died after 48 h, suggesting [^99m^Tc]Tc-tricine-TPPTS-HYNICNM was safe.

## 4. Materials and Methods

### 4.1. Materials 

All chemicals were of A. R. grade and used as received without further purification. Assignment of the spectra was based on ^1^H NMR, ^13^C NMR, IR, and HR-MS experiments, as previously published methods [36]. [^99m^Tc]NaTcO_4_ was obtained in a saline solution from a ^99m^Mo/^99m^Tc generator [45,46] supplied by the China Institute of Atomic Energy. The radioactivity of the samples was assessed by an HRS-1000 technetium analyzer and Wizard 2480 gamma counter. Reversed-phase HPLC analysis was performed on a Shimadzu HPLC pump and a Shimadzu UV absorbance detector. Kunming female mice (20 ± 2 g) and the murine sarcoma S180 cell line were obtained from the Peking University Health Science Center. SPECT/CT imaging was conducted by means of micro SPECT/CT equipment (Trifoil, CA, USA).

### 4.2. Synthesis of HYNICNM

The reaction routes of the ligand, HYNICNM, are shown in Scheme 1. The HYNIC-containing active ester (Compound 1) and the amino derivative of 2-nitroimidazole (Compound 2) were prepared according to previous literature [13,35] with slight modification, and the details are shown in the Supplementary Material.

Synthesis of HYNICNM. Compound 1 (0.440 g, 1 mmol), compound 2 (0.156 g, 1 mmol) and triethylamine (0.420 mL, 3 mmol) were dissolved in 10 mL of DMF. Then, the mixture was heated to reflux for 4 h. Afterward, the solvent was removed under reduced pressure, and the residue was purified by chromatography (CH_2_Cl_2_/CH_3_OH = 5:1) to obtain the ligand HYNICNM as a yellow solid (0.305 g, yield, 67%). ^1^H-NMR (400 MHz, D_2_O) δ(ppm): 8.61 (s, 1H), 8.23 (d, *J* = 2.4 Hz, 1H), 8.01 (d, *J* = 7.9 Hz, 1H), 7.80 (d, *J* = 7.5 Hz, 1H), 7.72 (dd, *J* = 8.9, 2.4 Hz, 1H), 7.48 (t, *J* = 7.6 Hz, 1H), 7.39 (t, *J* = 7.5 Hz, 1H), 7.34 (d, *J* = 1.2 Hz, 1H), 7.09 (d, *J* = 1.2 Hz, 1H), 7.00 (d, *J* = 8.9 Hz, 1H), 4.57 (t, *J* = 5.7 Hz, 2H), 3.66 (t, *J* = 5.3 Hz, 2H); ^13^C-NMR (100 MHz, D_2_O) δ(ppm): 168.23, 157.38, 147.08, 144.52, 140.53, 140.34, 137.36, 131.53, 131.25, 129.21, 128.47, 127.80, 126.86, 126.58, 120.19, 107.48, 49.44, 38.99; IR (KBr)/cm ^−1^:3384.15, 3367.86, 3317.71, 3277.20, 3269.48, 2918.42, 2848.98, 2378.33, 2345.54, 1637.63, 1608.84, 1533.47, 1489.11, 1473.68, 1398.45, 1363.73, 1190.13, 1128.41, 1018.46, 615.32; HR-MS (ESI) for C_18_H_16_N_7_O_6_S [M-Na]^−^: found 458.0890, calcd 458.0888.

### 4.3. Radiolabeling and Quality Control

Radiolabeling of [^99m^Tc]Tc-tricine-TPPTS-HYNICNM. HYNICNM ligand (1 mg, 2.08 ¦Ìmol) was dissolved in 0.5 mL of acetate buffer solution (pH = 5.0, 0.5 M). Then, TPPTS (1 mg, 1.76 μmol), tricine (8 mg, 44.65 μmol) and 0.04 mL (0.18 μmol) of SnCl_2_·2H_2_O (1 mg/mL,) were added to the solution. Next, 0.5 mL of freshly eluted [^99m^Tc]NaTcO_4_ (37–74 MBq) was added immediately. The mixture was heated at 100 °C for 30 min to produce [^99m^Tc]Tc-tricine-TPPTS-HYNICNM without further purification (radiochemical yield > 95%).

Radiolabeling of [^99m^Tc]Tc-tricine-TPPMS-HYNICNM. HYNICNM ligand (1 mg, 2.08 μmol) was dissolved in 0.5 mL of acetate buffer solution (pH = 5.0, 0.5 M). Then, TPPMS (1 mg, 2.74 μmol), tricine (8 mg, 44.65 μmol), and 0.04 mL of SnCl_2_·2H_2_O (1 mg/mL, 0.18 μmol) were added to the solution. Next, 0.5 mL of freshly eluted [^99m^Tc]NaTcO_4_ (37–74 MBq) was added immediately. The mixture was heated at 100 °C for 30 min to produce [^99m^Tc]Tc-tricine-TPPMS-HYNICNM without further purification (radiochemical yield > 95%).

Radiolabeling of [^99m^Tc]Tc-(tricine)_2_-HYNICNM. HYNICNM ligand (1 mg, 2.08 μmol) was dissolved in 0.5 mL of acetate buffer solution (pH = 5.0, 0.5 M). Then, tricine (8 mg, 44.65 μmol) and 0.04 mL of SnCl_2_·2H_2_O (1 mg/mL, 0.18 μmol) were added to the solution. Next, 0.5 mL of freshly eluted [^99m^Tc]NaTcO_4_ (37–74 MBq) was added immediately. The mixture was heated at 100 °C for 30 min to produce [^99m^Tc]Tc-(tricine)_2_-HYNICNM without further purification (radiochemical yield > 95%).

The radiochemical purity (RCP) of the technetium-99m complexes was determined by radio-HPLC and TLC. For radio-HPLC, a water (A) and methanol (B) mixture was adopted as the mobile phase with a flow rate of 1 mL/min, and the analytical conditions were as follows: 0 min 0% B, 5 min 0% B, 10 min 20% B, 20 min 50% B, and 25 min 0% B. For TLC, TLC was performed on a filter paper eluted with saline.

### 4.4. In Vitro Stability Study

To determine the in vitro stability, the technetium-99m complexes ([^99m^Tc]Tc-tricine-TPPTS-HYNICNM, [^99m^Tc]Tc-tricine-TPPMS-HYNICNM, and [^99m^Tc]Tc-(tricine)_2_-HYNICNM) were dissolved in saline at room temperature for 6 h, and the resulting solutions were checked by HPLC. For stability in mouse serum, the technetium-99m complexes (0.1 mL, 1.8 MBq) were incubated in mouse serum (0.1 mL) at 37 °C. After incubation for 6 h, 0.2 mL of acetonitrile was added to the sample, and the mixture was centrifuged at 6000× *g* for 5 min to precipitate the proteins. Afterward, the supernatant was filtered through a 0.22 μm filter and then analyzed by HPLC.

### 4.5. Determination of the Partition Coefficient (log P)

To measure the partition coefficient, 0.1 mL of radiolabeled complex(1.8 MBq) was mixed with 0.6 mL of phosphate buffer solution (0.025 M, pH 7.4) and 0.7 mL of 1-octanol in each centrifuge tube. After vortexing for 3 min and centrifugation at 10,000× *g* for 5 min, a clear separation of the two layers was obtained. Equal aliquots (0.1 mL) in triplicate were withdrawn from 1-octanol and phosphate buffer and measured in a γ-counter, and the readings were used to calculate the partition coefficient (log P). The value of P was determined as follows:

P = (counts in 1-octanol layer)/(counts in phosphate buffer layer).(1)

All experiments were performed in triplicate, and the log P values were expressed as the mean ± SD (standard deviation).

### 4.6. In Vitro Cellular Uptake

In vitro cellular uptake of the technetium-99m complexes was performed according to a previously reported method [47] with the murine sarcoma S180 cell line. Cells were suspended at a concentration of 2.0 × 10^6^ cells/mL with culture medium (20 mL), which was provided as DMEM (Dulbecco’s modified Eagle medium) containing 10% (v/v) FBS (fetal bovine serum). Then, the mixture was placed in glass vials with gentle stirring at 37.0 °C and incubated under hypoxic (95% N_2_ and 5% CO_2_) and aerobic (19% O_2_, 76% N_2_, and 5% CO_2_) conditions. Afterward, 0.2 mL (3.7 MBq) of the technetium-99m complexes were added to the vessels. One milliliter of the mixture was removed and centrifuged at 3000× *g* for 5 min at 0.5, 1, 2, and 4 h post-incubation, and then 0.9 mL of supernatant was taken for counting (A). The residual amount was designated as (B), and the cellular uptake was calculated as follows:

% uptake = (B − A/9)/(A + B).(2)

Each group was repeated five times, and the final results are expressed as the mean ± SD.

### 4.7. Biodistribution Studies

Animal studies were carried out in accordance with the principles of laboratory animal care and the guidelines of the Ethics Committee of Beijing Normal University (permit no. CLS-EAW-2018-001, June 2018). Kunming female mice (18–22 g) bearing S180 tumors were used in the biodistribution studies. About 1 × 10^6^ S180 cells were implanted in the left front armpit of female mice by subcutaneous injection. After one week, tumors grew to diameters of 5–8 mm. The mice were injected with a radiolabeled complex (0.1 mL, 740 kBq mL^−1^) via the tail vein and sacrificed at 0.5 h, 2 h, and 4 h post-injection. The organs and tissues of interest (tumor, blood, muscle, heart, liver, lung, kidney, spleen, stomach, bone, small intestine) were extracted, cleaned, weighed, and quantified for radioactivity. There were five mice in each group, and the final results are expressed as the percent uptake of injected dose per gram ± standard deviation (% IA/g ± SD).

### 4.8. SPECT/CT Imaging Studies

The SPECT/CT images were carried out according to a procedure described in our previous literature [48]. The radiolabeled complex (0.1 mL, 14.8 MBq) was administered via the tail vein in Kunming female mice bearing S180 tumor, and the mice were sacrificed at 2 h post-injection. SPECT/CT imaging data were collected by a micro SPECT/CT scanner with HiSPECT software and Vivoquant 2.5 software. 

## 5. Conclusions

In this study, a novel 2-nitroimidazole HYNIC derivative (HYNICNM) was synthesized and radiolabeled with technetium-99m using tricine/TPPTS, tricine/TPPMS, and tricine to produce [^99m^Tc]Tc-tricine-TPPTS-HYNICNM, [^99m^Tc]Tc-tricine-TPPMS-HYNICNM, and [^99m^Tc]Tc-(tricine)_2_-HYNICNM, respectively. These complexes all exhibited good in vitro stability and hypoxic selectivity. The co-ligands had a significant effect on the hydrophilicity and biodistribution properties of the technetium-99m complexes. Among them, [^99m^Tc]Tc-tricine-TPPTS-HYNICNM exhibited the highest tumor uptake, most rapid clearance from the liver, blood, muscle, and the highest tumor/non-target uptake. Moreover, as [^99m^Tc]Tc-tricine-TPPTS-HYNICNM can be prepared in one pot in one step, it can be generated as a kit formulation; therefore, it is a promising candidate for imaging tumor hypoxia in the clinic.

## Figures and Tables

**Table 1:** Biodistribution of [^99m^Tc]Tc-tricine-TPPTS-HYNICNM in Kunming female mice bearing S180 tumors at 30 min, 120 min, and 240 min post-injection (% IA/g ± s, *n* = 5).

Tissue	30 min	120 min	240 min
Heart	1.64 ± 0.18	0.56 ± 0.04	0.45 ± 0.09
Liver	1.30 ± 0.09	0.54 ± 0.09	0.50 ± 0.06
Lung	2.49 ± 0.25	0.62 ± 0.07	0.48 ± 0.06
Kidney	5.49 ± 0.27	3.77 ± 0.39	3.93 ± 0.49
Spleen	0.78 ± 0.12	0.28 ± 0.05	0.27 ± 0.05
Stomach	0.79 ± 0.17	0.57 ± 0.24	0.55 ± 0.18
Bone	0.60 ± 0.16	0.39 ± 0.03	0.38 ± 0.03
Muscle	0.85 ± 0.10	0.21 ± 0.01	0.20 ± 0.02
Small Intestine	1.83 ± 0.34	0.50 ± 0.06	0.43 ± 0.11
Tumor	2.04 ± 0.22	1.05 ± 0.27	0.84 ± 0.24
Blood	3.51 ± 0.20	0.73 ± 0.04	0.50 ± 0.05
Thyroid(%IA)	0.06 ± 0.01	0.02 ± 0.01	0.03 ± 0.01
Tumor/Liver	1.56	1.95	1.66
Tumor/Blood	0.58	1.44	1.68
Tumor/Muscle	2.41	5.05	4.10

## Data Availability

The data presented in this study are available in this article.

## Supplementary Materials

The following are available online at https://www.mdpi.com/1424-8247/14/2/158/s1, synthesis of Compound **1** and Compound **2**, Figure S1: ^1^H NMR spectrum of Compound **1**, Figure S2: ^1^H NMR spectrum of Compound **2**, Figure S3: ^1^H NMR spectrum of HYNICNM, Figure S4: ^13^C NMR spectrum of HYNICNM, Figure S5: IR spectrum of HYNICNM, Figure S6: HR-MS spectrum of HYNICNM, Figure S7: HPLC patterns of [^99m^Tc]Tc-Tricine and [^99m^Tc]Tc-Tricine-TPPTS, Figure S8: In vitro stability HPLC profiles of [^99m^Tc]Tc-tricine-TPPTS-HYNICNM, Figure S9: In vitro stability HPLC profiles of [^99m^Tc]Tc-tricine-TPPMS-HYNICNM, Figure S10: In vitro stability HPLC profiles of [^99m^Tc]Tc-(tricine)_2_-HYNICNM, Table S1: Biodistribution of [^99m^Tc]Tc-tricine-TPPTS-HYNICNM, [^99m^Tc]Tc-tricine-TPPMS-HYNICNM, and [^99m^Tc]Tc-(tricine)_2_-HYNICNM.
