# Supplementary material for: Preparation and Bioevaluation of Novel 99mTc-Labeled Complexes with a 2-Nitroimidazole HYNIC Derivative for Imaging Tumor Hypoxia
Source: Pharmaceuticals (Basel). 2021 Feb 15;14(2):158. doi: 10.3390/ph14020158 (PMC7919024; doi:10.3390/ph14020158)
Supplement: Supplementary file 1 [file pharmaceuticals-14-00158-s001.pdf]

Supplementary data

Preparation and bioevaluation of novel ^{99m}Tc-labelled complexes with a 2-nitroimidazole HYNIC derivative for imaging tumor hypoxia

Qing Ruan, Qianqian Gan, Xuran Zhang, Si'an Fang, Junbo Zhang*

Key Laboratory of Radiopharmaceuticals, Ministry of Education, College of Chemistry, Beijing Normal University, Beijing, 100875, P. R. China.

* Corresponding Author: No. 19 Xinjiekou Wai Boulevard, Haidian District, Beijing. College of Chemistry, Beijing Normal University, 100875, P. R. (China). E-mail: zhjunbo@bnu.edu.cn

Content

Synthesis of Compound 1 and Compound 2. .... S3

Figure S1. ^{1}H NMR spectrum of Compound 1. .... S4

Figure S2. ^{1}H NMR spectrum of Compound 2. .... S4

Figure S3. ^{1}H NMR spectrum of HYNICNM. S5

Figure S4. ^{13}C NMR spectrum of HYNICNM. S5

Figure S5. IR spectrum of HYNICNM........ S6

Figure S6. HR-MS spectrum of HYNICNM. S6

Figure S7. HPLC patterns of [^{99m}Tc]Tc-tricine and [^{99m}Tc]Tc-tricine-TPPTS........ S7

Figure S8. *In vitro* stability HPLC profiles of [^{99m}Tc]Tc-tricine-TPPTS-HYNICNM..... S7

Figure S9. *In vitro* stability HPLC profiles of [^{99m}Tc]Tc-tricine-TPPMS-HYNICNM.... S8

Figure S10. *In vitro* stability HPLC profiles of [^{99m}Tc]Tc-(tricine)$_2$-HYNICNM S8

Table S1. Biodistribution of [^{99m}Tc]Tc-tricine-TPPTS-HYNICNM, [^{99m}Tc]Tc-tricine-TPPMS-HYNICNM and [^{99m}Tc]Tc-(tricine)$_2$-HYNICNM S9

Synthesis of Compound 1

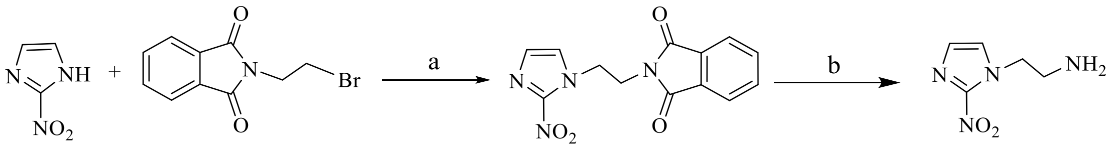

5 4 3 1

Scheme S1. The synthesis of Compound 1. Reagents and conditions: (a) DMF, K_2CO_3, reflux, 3 h; (b) EtOH, hydrazine hydrate, reflux, 4 h.

Compound 5 (2-nitro-1*H*-imidazole, 1.13 g, 10 mmol) and Compound 4 (2-(2-bromoethyl)isoindoline-1,3-dione, 3.05 g, 12 mmol) were added into DMF (50 mL). After an addition of K_2CO_3 (0.98 g, 12 mmol), the mixture was refluxed for 3 h at 110 °C. The solvent was cooled to the room temperature before the solid was filtered from the solution. Then the solid was washed with water and dried to give crude product (compound 3, 2-(2-(2-nitro-1*H*-imidazol-1-yl)ethyl)isoindoline-1,3-dione, 2.56 g, 89 %).

Compound 3 (1.43 g, 5 mmol) and hydrazine hydrate (0.50 g, 10 mmol) were dissolved in ethanol (50 mL). Then the mixture was refluxed for 4 h. After cooled to the room temperature, the mixture was stored into refrigerator at 0°C for 12 h. After the insoluble substance was removed by filtration, the solvent was removed under a reduced pressure and the residue was purified by chromatography (CH_2Cl_2/CH_3OH) to give Compound 1 (2-(2-nitro-1*H*-imidazol-1-yl)ethan-1-amine, 0.61 g, 77%). ^{1}H-NMR (400 MHz, D_2O): ^{1}H NMR (600 MHz, Deuterium Oxide) δ 8.05 (s, 1H), 7.60 (s, 1H), 4.06 (t, *J* = 6.0 Hz, 2H), 2.96 (t, *J* = 5.9 Hz, 2H); MS (m/z): $[M + H]^+$ 157.01。

Synthesis of Compound 2

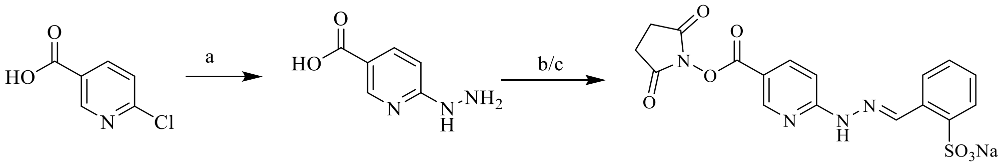

7 **6** **2**

Scheme S2. The synthesis of Compound 2. Reagents and conditions: (a) hydrazine hydrate, 100 °C, 4 h; (b) sodium 2-formylbenzenesulfonate, r.t., 3 h; (c) *N*-hydroxysuccinimide, DCC, r.t., 18 h; reflux, 1 h.

Compound 7 (6-chloronicotinic acid, 1.57 g, 10 mmol) was dissolved in 80% hydrazine hydrate (10 mL). Then, the mixture was stirred at 100 ℃ for 4 h. After cooling to room temperature, solvents were removed under reduced pressure. Water (20 mL) was added to the residue and the pH was adjusted to 5.0 with concentrated hydrochloric acid, after which a precipitate was formed. Filtration was then performed. The filter cake was washed with 95% ethanol and ether and dried in vacuo to give a yellow solid (Compound 6, 6-hydrazinylnicotinic acid, 1.30 g, 85%).

2-formylbenzenesulfonic acid sodium (0.87 g, 4 mmol) was added to a suspension of Compound 6 (1.28 g, 8 mmol) in DMF (10 mL), and the reaction mixture was allowed to stir at room temperature for 3 h. Then, *N*-hydroxysuccinimide (0.46 g, 4 mmol) and *N*,*N'*-Dicyclohexylcarbodiimide (1.60 g, 8 mmol) were added to the above mixture. After stirring at room temperature for another 18 h, the reaction mixture was filtered and the filtrate was concentrated. Ethyl acetate (50 mL) was added to the residue and the mixture was heated to reflux for 1 h, after which filtration was carried out while it was hot. A light yellow solid (Compound 2, sodium (E)-2-((2-(5-(((2,5-dioxopyrrolidin-1-yl)oxy)carbonyl)pyridin-2-yl) hydrazono) methyl) benzenesulfonate) was finally obtained after drying the filter cake in vacuo (1.33 g, yield 76%).^{1}H NMR (600 MHz, DMSO-d_6) δ 11.86 (s,

1H), 9.13 (s, 1H), 8.76 (d, J = 2.3 Hz, 1H), 8.14 (dd, J = 9.0, 2.4 Hz, 1H), 8.00 (dd, *J* = 7.6, 1.5 Hz, 1H), 7.77 (dd, *J* = 7.6, 1.6 Hz, 1H) 7.36 – 7.31 (m, 3H), 2.69 (s, 4H). MS (*m/z*): $[M\text{-}Na]^-$ 417.18.

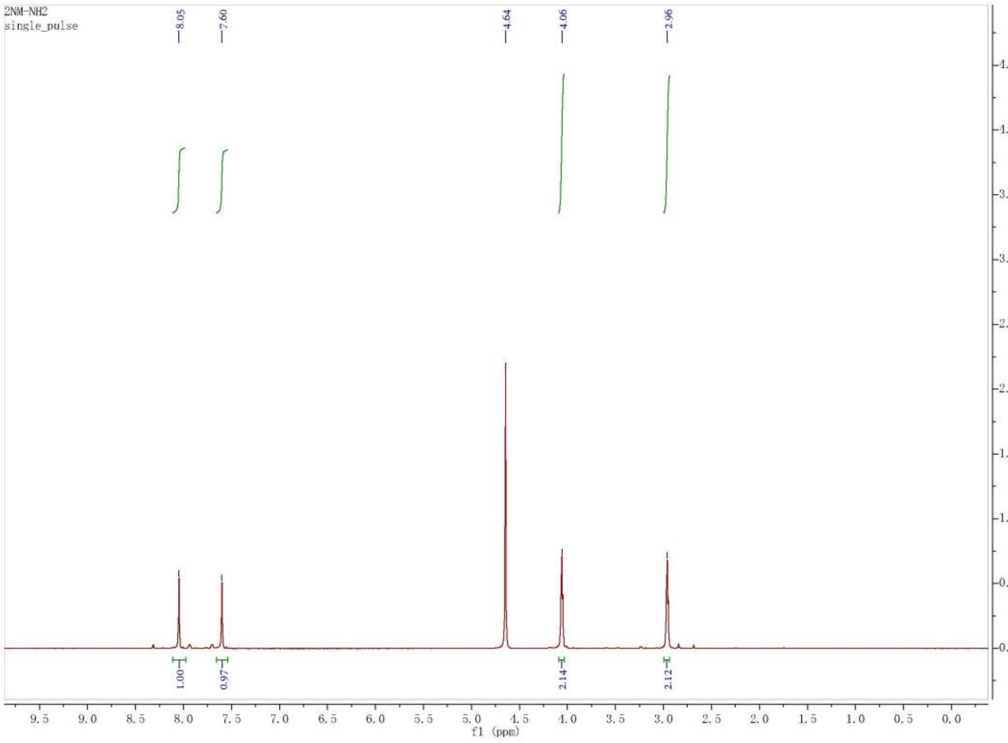

Figure S1. ^{1}H NMR spectrum of Compound 1

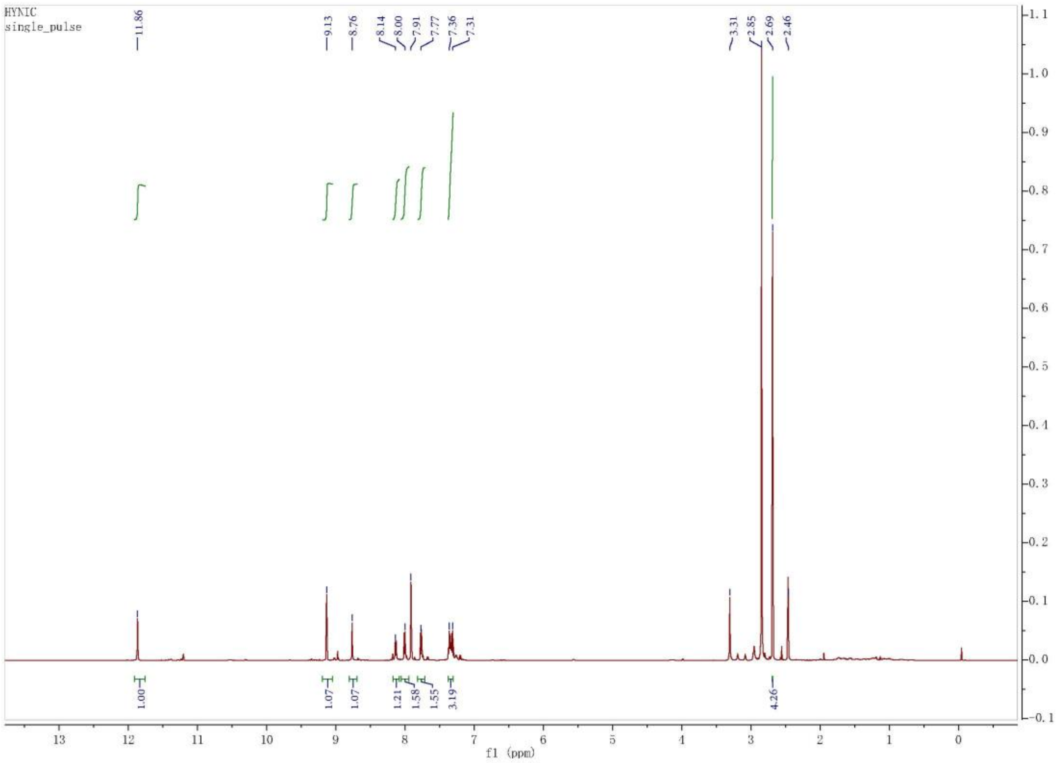

Figure S2. ^{1}H NMR spectrum of Compound 2

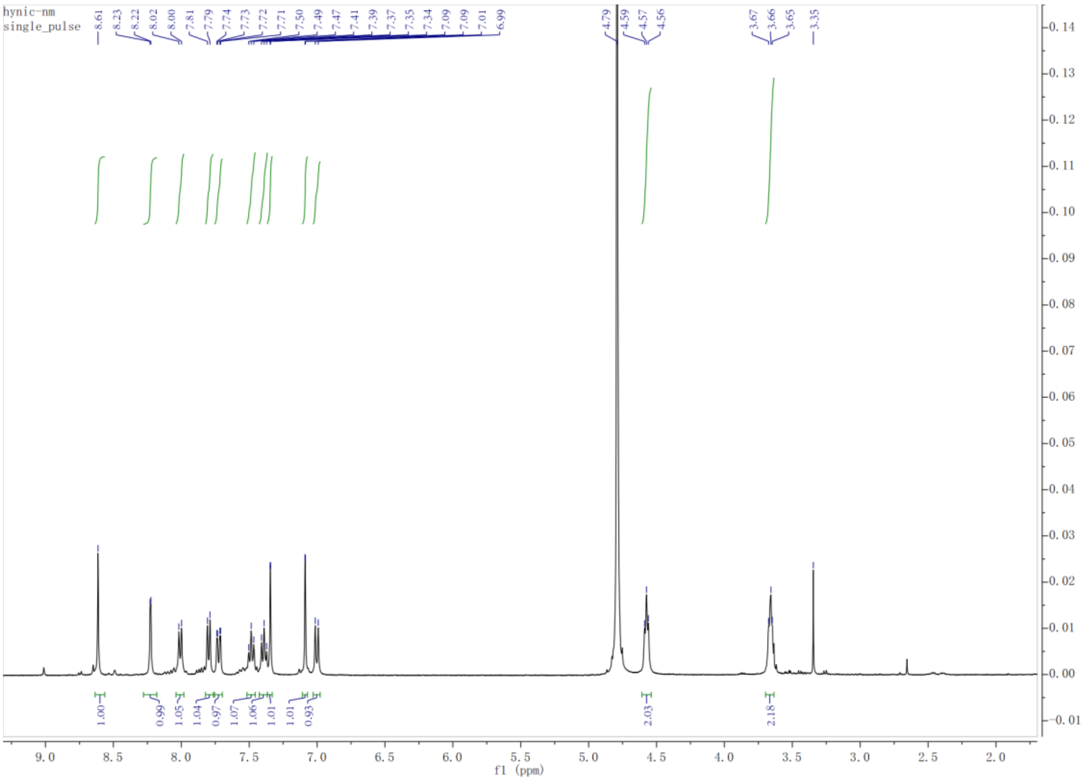

Figure S3. ^{1}H NMR spectrum of HYNICNM

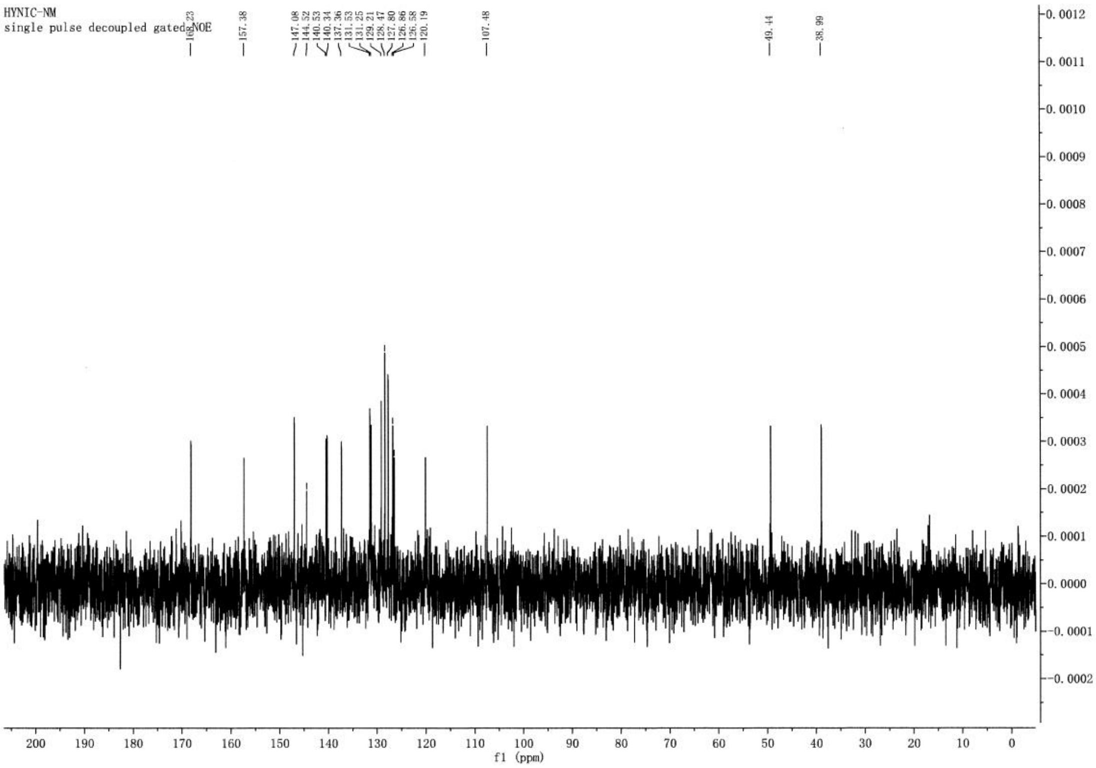

Figure S4. ^{13}C NMR spectrum of HYNICNM

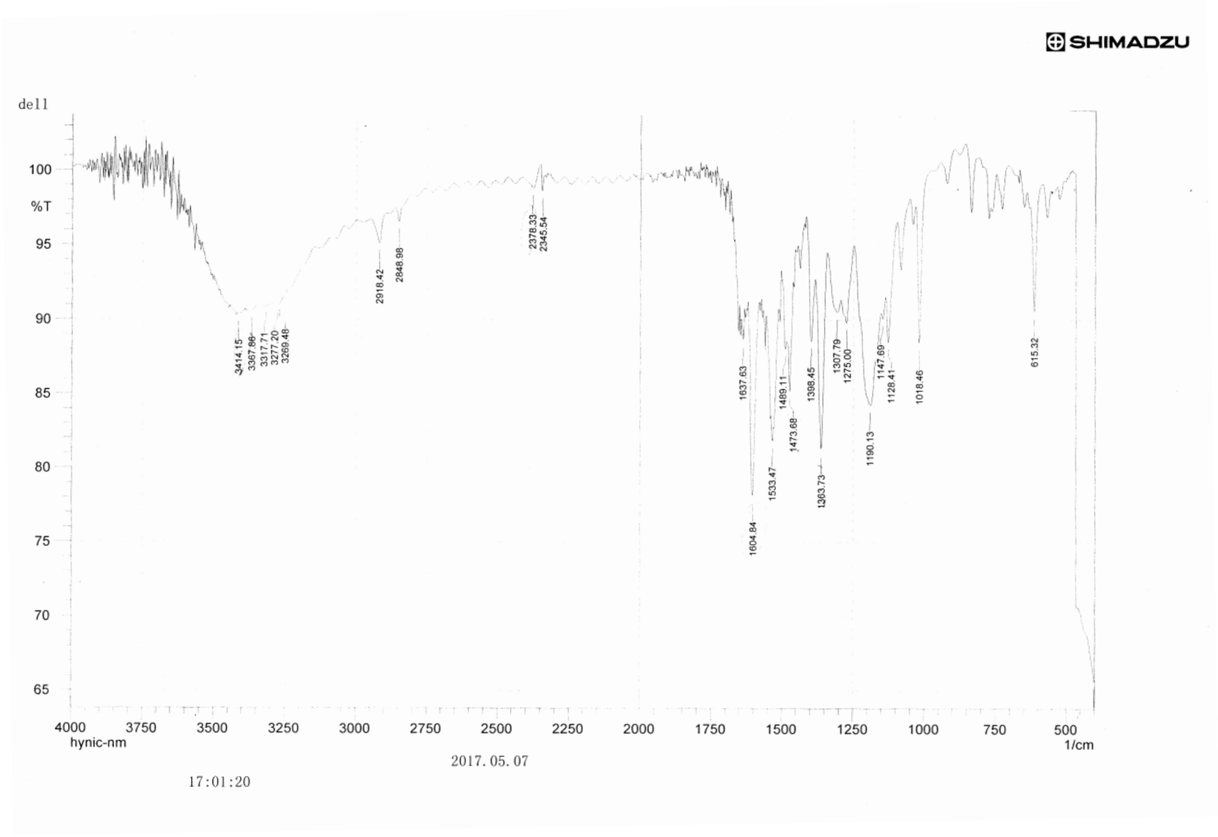

Figure S5. IR spectrum of HYNICNM

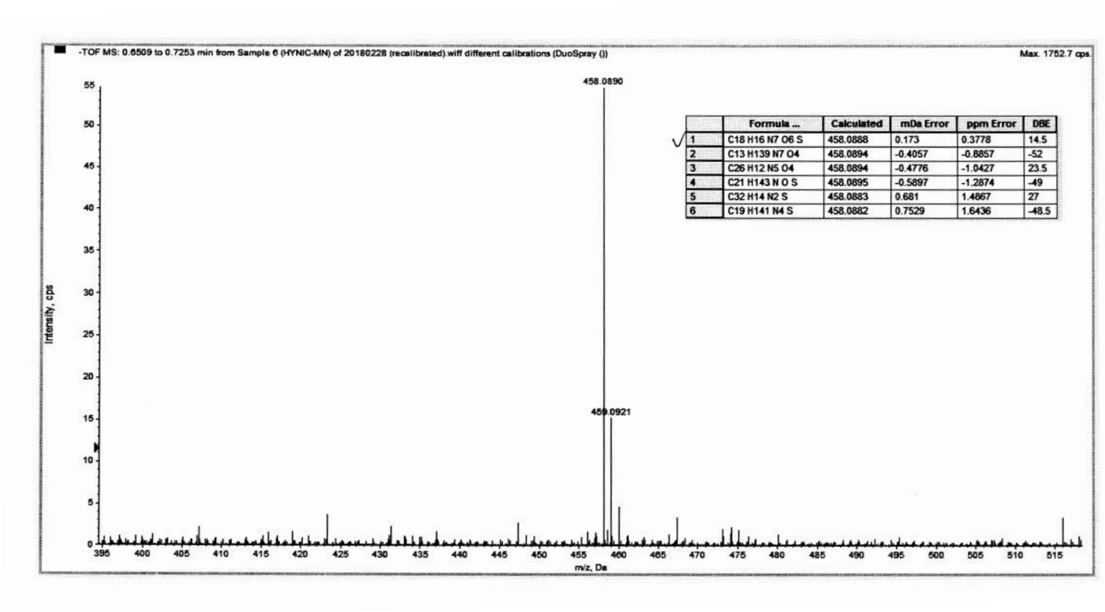

Figure S6. HR-MS spectrum of HYNICNM

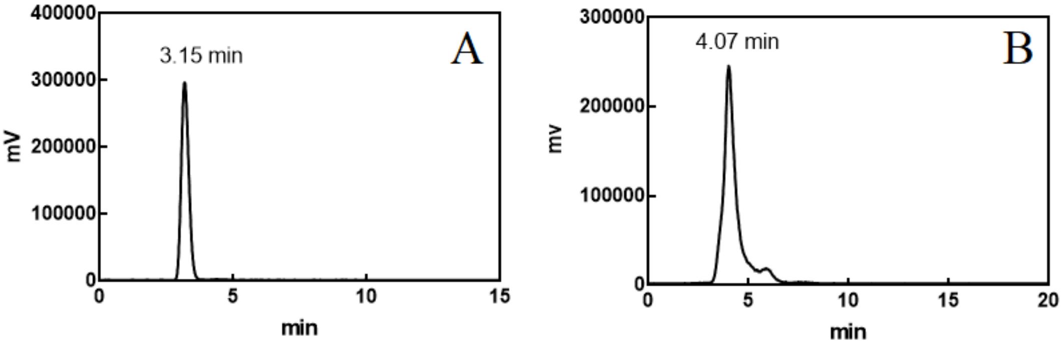

Figure S7. HPLC patterns of [^{99m}Tc]Tc-Tricine (A) and [^{99m}Tc]Tc-tricine-TPPTS(B).

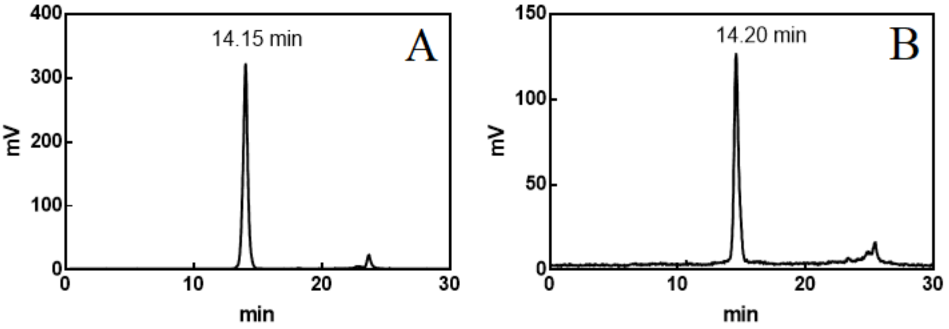

Figure S8. *In vitro* stability HPLC profiles of [^{99m}Tc]Tc-tricine-TPPTS-HYNICNM in saline (A) and in mouse serum (B) for 6 h.

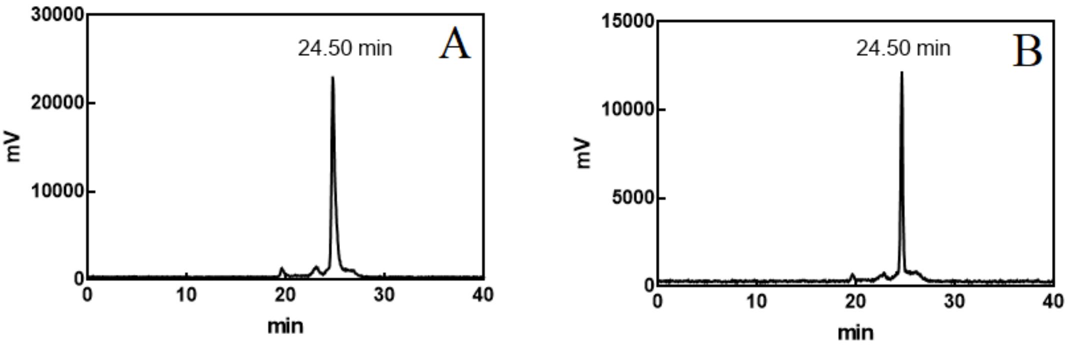

Figure S9. *In vitro* stability HPLC profiles of [^{99m}Tc]Tc-tricine-TPPMS-HYNICNM in saline (A) and in mouse serum (B) for 6 h.

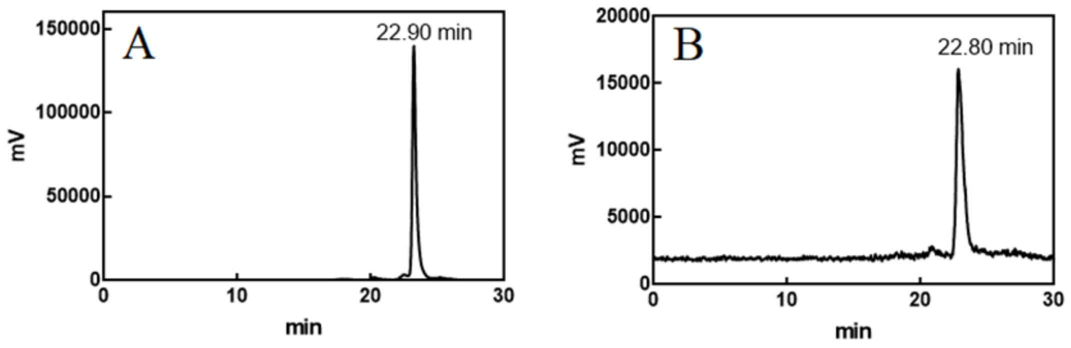

Figure S10. *In vitro* stability HPLC profiles of [^{99m}Tc]Tc-(tricine)$_2$-HYNICNM in saline (A) and in mouse serum (B) for 6 h.

Table S1. Biodistribution of [^{99m}Tc]Tc-tricine-TPPTS-HYNICNM, [^{99m}Tc]Tc-tricine-TPPMS-HYNICNM and [^{99m}Tc]Tc-(tricine)$_2$-HYNICNM in Kunming female mice bearing S180 tumor at 2 h post-injection (%IA/g ± s, n=5).

Tissue	[^{99m}Tc]Tc-tricine-TPPTS-HYNICNM	[^{99m}Tc]Tc-tricine-TPPMS-HYNICNM	[^{99m}Tc]Tc-(tricine)$_2$-HYNICNM
Heart	0.56±0.04	0.23±0.05	0.31±0.04
Liver	0.54±0.09	0.59±0.10	1.52±0.38
Lung	0.62±0.07	0.29±0.05	0.68±0.08
Kidney	3.77±0.39	0.86±0.10	2.10±0.46
Spleen	0.28±0.05	0.30±0.07	0.20±0.04
Stomach	0.57±0.24	0.32±0.14	1.02±0.40
Bone	0.39±0.03	0.41±0.14	0.23±0.03
Muscle	0.21±0.01	0.18±0.04	0.15±0.03
Small Intestine	0.50±0.06	1.02±0.06	1.61±0.56
Tumor	1.05±0.27	0.27±0.06	0.62±0.20
Blood	0.73±0.04	0.23±0.01	0.77±0.09
Thyroid(%IA)	0.02±0.01	0.02±0.00	0.01±0.01
Tumor/Liver	1.95	0.45	0.41
Tumor/Blood	1.44	1.15	0.81
Tumor/Muscle	5.05	1.50	4.08
